# Diagnostic accuracy of ultrasound shear wave elastography combined with superb microvascular imaging for breast tumors: A protocol for systematic review and meta-analysis

**DOI:** 10.1097/MD.0000000000026262

**Published:** 2021-06-25

**Authors:** Jinyi Bian, Jili Zhang, Xiukun Hou

**Affiliations:** a The First Affiliated Hospital of Dalian Medical University; b Ultrasound department of the First Affiliated Hospital of Dalian Medical University, Dalian, China.

**Keywords:** breast tumors, meta-analysis, shear wave elastography, superb microvascular imaging

## Abstract

**Background:**

Shear wave elastography (SWE) is a new ultrasonic elastography technique for evaluating the hardness of living tissue by measuring the propagation velocity of shear wave in tissue, which is characterized by real-time, non-invasive and quantitative. The SWE technique can be used to diagnose the lesions of different tissues and organs, and the quantitative measurement of SWE is considered as more objective information about breast masses. Superb microvascular imaging (SMI) is a new noninvasive Doppler ultrasound imaging method, which can display blood flow information with high spatial resolution and high frame rate, while keeping the minimum low-speed blood flow components. Therefore, SMI can diagnose diseases closely related to angiogenesis at a relatively early stage. However, the results of these studies have been contradictory. The present meta-analysis aimed at determining the accuracy of SWE combined with SMI in the differential diagnosis between benign and malignant breast lesions.

**Methods:**

We will search PubMed, Web of Science, Cochrane Library, and Chinese biomedical databases from their inceptions to the April 18, 2021, without language restrictions. Two authors will independently carry out searching literature records, scanning titles and abstracts, full texts, collecting data, and assessing risk of bias. Review Manager 5.2 and Stata14. 0 software will be used for data analysis.

**Results:**

This systematic review will determine the accuracy of shear wave elastography combined with superb microvascular imaging in the differential diagnosis between benign and malignant breast tumors.

**Conclusion:**

Its findings will provide helpful evidence for the accuracy of shear wave elastography combined with superb microvascular imaging in the differential diagnosis between benign and malignant breast tumors.

**Systematic review registration:**

INPLASY202150075

## 1 Introduction

Breast cancer is one of the most common malignant tumors of women in the 21st century, and it is also the main cause of cancer-related deaths for women in the world. The early manifestation of breast cancer is atypical and easy to be misdiagnosed or missed. Thus, how to accurately distinguish benign and malignant breast lesions is the focus of the development of breast ultrasound technology.^[1,2]^ In breast tumors, tissue hardness is related to the risk of malignant tumor: the harder the tumor, the higher the risk of malignant tumor. The basic principle of Shear wave elastography is that the vibration of tissue particles is caused by the acoustic radiation pulse generated by focused ultrasonic beam, displacement is induced at the focus and transverse shear wave is generated. The shear wave velocity (SWV) of the detected tissue can be measured accurately and quantitatively, and the SWV can be color-coded and superimposed on the 2-dimensional anatomical image, thus forming a real-time shear wave velocity map of living tissue. Therefore, shear wave elastography (SWE) can be used to diagnose the lesions of different tissues and organs, and the quantitative measurement of SWV is considered as more objective information about breast masses.^[3–5]^ However, there is no uniform standard for the elastic parameters and their limits in diagnosing benign and malignant breast lesions, and more clinical research is needed. Tumor angiogenesis plays an important role in the development, growth, and metastasis of breast cancer, and the number of microvessels in the lesion is closely related to the malignancy of the tumor.^[6]^ Superb microvascular imaging (SMI) is a new noninvasive Doppler ultrasound imaging method, which uses a new clutter suppression algorithm to identify and eliminate the movement of tissue itself, extract blood flow signals at a relatively high frame rate, and provide high-resolution details of vascular branches without ultrasound contrast agent. SMI can display blood flow information with high spatial resolution and high frame rate, while keeping the minimum low-speed blood flow components so that diagnoses diseases closely related to angiogenesis at a relatively early stage.^[7–9]^

Studies indicate that SWE combined with SMI is of great value in differential diagnosis of benign and malignant breast masses.^[10]^ However, the results of these studies have been contradictory. Thus, the current meta-analysis aims at determining the accuracy of SWE combined with SMI in the differential diagnosis between benign and malignant breast lesions to provide reference for the diagnosis and clinical treatment of breast cancer.

## 2 Materials and methods

This study was conducted in accordance with the PRISMA (Preferred Reporting Items for Systematic Reviews and Meta Analyses) guidelines and the protocol was registered in the INPLASY (INPLASY202150075).

### 2.1 Eligibility criteria

#### 2.1.1 Type of study

This study will only include high-quality clinical cohort or case–control studies.

#### 2.1.2 Type of patients

The patients should be those who had undergone breast tumors.

#### 2.1.3 Intervention and comparison

This study compared the diagnostic value of SWE combined with SMI and pathology in breast tumors.

#### 2.1.4 Type of outcomes

The primary outcomes include sensitivity, specificity, positive and negative likelihood ratio, diagnostic odds ratio, and the area under the curve of the summary receiver-operating characteristic.

### 2.2 Search methods

PubMed, Web of Science, Cochrane Library, and Chinese biomedical databases will be searched from their inceptions to May 18, 2021, without language restrictions. The search strategy for PubMed is shown in Table 1. Other online databases will be used in the same strategy.

**Table 1:** Search strategy sample of PubMed.

Number	Search terms
1	Breast tumors, breast lesions, or breast cancer.
2	Pathology
3	Shear wave elastography or SWE
4	Superb microvascular imaging or SMI

### 2.3 Data extraction and quality assessment

Two authors will independently select the trials according to the inclusion criteria, and import into Endnote X9. Then remove duplicated or ineligible studies. Screen the titles, abstracts, and full texts of all literature to identify eligible studies. All essential data will be extracted using previously created data collection sheet by 2 independent authors. Discrepancies in data collection between 2 authors will be settled down through discussion with the help of another author. The following data will be extracted from each included research: the first author's surname, publication year, language of publication, study design, sample size, number of lesions, source of the subjects, instrument, “criterion standard,” and diagnostic accuracy. The true positives, true negatives, false positives, and false negatives in the 4-fold (2 × 2) tables were also collected. Methodological quality was independently assessed by 2 researchers based on the quality assessment of studies of diagnostic accuracy studies (QUADAS) tool. The QUADAS criteria included 14 assessment items. Each of these items was scored as “yes” (2), “no” (0), or “unclear” (1). The QUADAS score ranged from 0 to 28, and a score ≥22 indicated good quality. Any disagreements between 2 investigators will be solved through discussion or consultation by a 3rd investigator.^[11]^

### 2.4 Statistical analysis

The STATA version 14. 0 (Stata Corp, College Station, TX) and Meta-Disc version 1. 4 (Universidad Complutense, Madrid, Spain) soft wares were used for meta-analysis. We calculated the pooled summary statistics for sensitivity, specificity, positive and negative likelihood ratio, and diagnostic odds ratio with their 95% confidence intervals. The summary receiver operating characteristic curve and corresponding area under the curve were obtained. The threshold effect was assessed using Spearman correlation coefficients. The Cochrans Q-statistic and *I* test were used to evaluate potential heterogeneity between studies. If significant heterogeneity was detected (Q test *P* < . 05 or *I* test >50%), a random-effects model or fixed-effects model was used.^[12]^ We also performed subgroup and meta-regression analyses to investigate potential sources of heterogeneity. To evaluate the influence of single studies on the overall estimate, a sensitivity analysis was performed. We conducted Beggs funnel plots and Eggers linear regression tests to investigate publication bias.

### 2.5 Ethics and dissemination

We will not obtain ethic documents because this study will be conducted based on the data of published literature. We expect to publish this study in a peer-reviewed journal.

## 3 Discussions

At present, high-frequency ultrasound is a cost-effective and widely used breast cancer screening tool which detects tumors through sound waves reflected from breast tissues. However, this technique is subjective and ambiguous, with high sensitivity and low specificity in differentiating benign and malignant breast cancer, and is easy to be misdiagnosed and missed in atypical lesions.^[13]^ Generally speaking, the hardness of breast tissue is malignant, benign, and normal in order from large to small. Young's modulus is a physical quantity to evaluate the elasticity of tissue, which is proportional to the square of the measured tissue density and the propagation velocity of the shear wave. Studies have shown that the Young's modulus of malignant breast lesions is higher than that of benign breast lesions. SWE can measure Young's modulus to analyze the elastic heterogeneity of breast lesions, and then distinguish benign and malignant lesions. Therefore, SWE can achieve acoustic palpation, objectively and quantitatively evaluate the hardness of lesions, and provide sufficient information for the differential diagnosis of benign and malignant lesions of breast cancer.^[14,15]^ The vascular morphology and distribution characteristics of tumor are closely related to the nature of tumor, which is an important basis for differentiating benign and malignant tumors. Specifically, malignant breast tumors secrete related growth factors to accelerate the formation of new blood vessels, so the shape of blood vessels is irregular and uneven. The venous return and lymphatic reticulum of benign breast tumors are in normal state, so the blood vessels walk naturally and are of equal thickness. Therefore, the state of tumor microvessels can be displayed by SMI, and the Adler method can be used to observe and evaluate the blood supply of the tumor from a certain plane to qualitatively analyze the benign and malignant breast tumors.^[16]^

Compared with conventional ultrasound, SWE provides a standard quantitative result for assessing tissue hardness, and SMI shows more subtle blood flow information in benign and malignant breast masses. These studies suggest that combining all quantitative values of SWE and SMI with B-type ultrasound can improve the diagnostic ability of benign and malignant breast lesions. To clarify this point, we will conduct a systematic review, summarizing high-quality studies and providing evidence-based medicine to support clinical practice.

## Author contributions

**Conceptualization:** Xiukun Hou.

**Data curation:** Jinyi Bian and Jili Zhang.

**Formal analysis:** Jinyi Bian, Jili Zhang.

**Funding acquisition:** Jinyi Bian, Jili Zhang.

**Investigation:** Jinyi Bian, Jili Zhang.

**Methodology:** Jinyi Bian and Jili Zhang.

**Project administration:** Jinyi Bian, Jili Zhang.

**Resources:** Jinyi Bian, Jili Zhang.

**Software:** Jinyi Bian, Jili Zhang.

**Supervision:** Jinyi Bian, Jili Zhang.

**Validation:** Jinyi Bian, Jili Zhang.

**Visualization:** Jinyi Bian, Jili Zhang.

**Writing – original draft:** Jinyi Bian and Jili Zhang.

**Writing – review & editing:** Jinyi Bian, Jili Zhang, and Xiukun Hou.
